# Retrospective seroepidemiology indicated that human enterovirus 71 and coxsackievirus A16 circulated wildly in central and southern China before large-scale outbreaks from 2008

**DOI:** 10.1186/1743-422X-7-300

**Published:** 2010-11-04

**Authors:** Zhen Zhu, Shuangli Zhu, Xuebin Guo, Jitao Wang, Dongyan Wang, Dongmei Yan, Xiaojuan Tan, Liuying Tang, Hui Zhu, Zhaohui Yang, Xiaohong Jiang, Yixin Ji, Yong Zhang, Wenbo Xu

**Affiliations:** 1 State Key Laboratory for Molecular Virology & Genetic Engineering, Institute for Viral Disease Control and Prevention, Chinese Center for Disease Control and Prevention, No.155, Changbai Road, Changping District, Beijing 102206, China; 2 Qinghai Center for Disease Control and Prevention, No. 66, Bayizhong Road, Xining 810007, China; 3 Taiyuan City Center for Disease Control and Prevention, No. 89, Xinjiannan Road, Taiyuan 030012, China; 4 Lanzhou University Second Hospital, No. 82, Cuiyingmen Road, Chengguan District, Lanzhou 730000, China

## Abstract

**Background:**

Large nationwide outbreaks of hand, foot, and mouth disease (HFMD) occurred in China from 2008; most of the cases were in children under 5 years. This study aims to identify the situation of natural human enterovirus 71 (HEV71) and coxsackievirus A16 (CVA16) infections in children before 2008 in China.

**Results:**

Retrospective seroepidemiologic studies of HEV71 and CVA16 were performed with 900 serum samples collected from children ≤5 years of age in 2005. The samples were collected from 6 different geographical areas (Anhui, Guangdong, Hunan, Xinjiang, Yunnan, and Heilongjiang provinces) in mainland China. Of the 900 samples, 288 were positive for HEV71; the total positive rate was 32.0% and the geometric mean titer (GMT) was 1:8.5. Guangdong (43.7% and 1:10.8), Xinjiang (45.4% and 1:11.1), and Yunnan (43.4% and 1:12.0) provinces had relatively high rates of infection, while Heilongjiang province (8.1% and 1:4.9) had the lowest rate of infection. On the other hand, 390 samples were positive for CVA16; the total positive rate was 43.4% and the GMT was 1:9.5. Anhui (62.2% and 1:16.0) and Hunan (61.1% and 1:23.1) had relatively high rates, while Heilongjiang (8.0% and 1:4.6) had the lowest rate. Although there is a geographical difference in HEV71 and CVA16 infections, low neutralizing antibody positive rate and titer of both viruses were found in all 6 provinces.

**Conclusions:**

This report confirmed that HEV71 and CVA16 had wildly circulated in a couple provinces in China before the large-scale outbreaks from 2008. This finding also suggests that public health measures to control the spread of HEV71 and CVA16 should be devised according to the different regional characteristics.

## Background

Hand, foot, and mouth disease (HFMD) was first reported in New Zealand in 1957. Coxsackievirus A16 (CVA16) and human enterovirus 71 (HEV71), which were first isolated in Canada and USA in 1958 and 1969, respectively, are the two major causative agents of HFMD. The co-circulation of both pathogens has been described previously [1-3]. HFMD is a common infectious disease in young children, particularly in those under 5 years. The disease is typically characterized by mucocutaneous papulovesicular rashes on hands, feet, mouth, and buttocks, and the infection usually occurs as outbreaks. HFMD usually resolves spontaneously. CVA16-associated HFMD has a milder outcome, with much lower incidence of severe complications, including death [4]. In contrast, a variety of neurological diseases, including aseptic meningitis, encephalitis, and poliomyelitis-like paralysis, can sometimes develop, particularly when HEV71 is the causative agent [5-8].

In recent years, numerous large outbreaks of HFMD have occurred in eastern and southeastern Asian countries and regions, including Singapore [6], South Korea [9], Malaysia [10], Japan [11], Vietnam [12], mainland China [2,13], and Taiwan [14,15]. HFMD was first reported in mainland China in 1981 and thereafter reported in most of the provinces of China. CVA16 was isolated in stool specimens of HFMD patients in Xiamen City in 1983, and HEV71 was first isolated in clinical specimens of HFMD patients in Wuhan City in 1987 [16]. Since the epidemic developed over a relatively short time span, HEV71-associated HFMD received considerable attention from clinicians and public health officials, and HFMD was classified as a category C notifiable infectious disease (In the notifiable infectious disease reporting system in China, total 39 kinds of infectious disease should be reported and be classified as three categories including A, B and C based on their epidemic situation and harmful degree, etc. Usually the harmful degree of category C diseases was less than category A and B diseases) by the Ministry of Health of China on May 2, 2008.

Large nationwide HFMD outbreaks have occurred in China since 2008, and most of the HFMD cases in these outbreaks were in children ≤5 years [17]. However, the epidemicity of HFMD before 2008 has not been well studied, and the disease surveillance system for HFMD has not been well established. To investigate the seroepidemiology of HFMD infection in China and devise appropriate preventive measures, retrospective seroepidemiologic studies of HEV71 and CVA16 were performed with serum samples collected during 2005 in 6 different geographical areas (Anhui, Guangdong, Heilongjiang, Hunan, Xinjiang, and Yunnan provinces) in mainland China.

## Results

### Geographical difference in HEV71 and CVA16 infections

Among the 900 serum samples surveyed, 288 were positive for HEV71, with a total positive rate of 32.0% and GMT of 1:8.5. On the other hand, 390 samples were positive for CVA16, with a total positive rate of 43.4% and GMT of 1:9.5.

**Figure 1:**
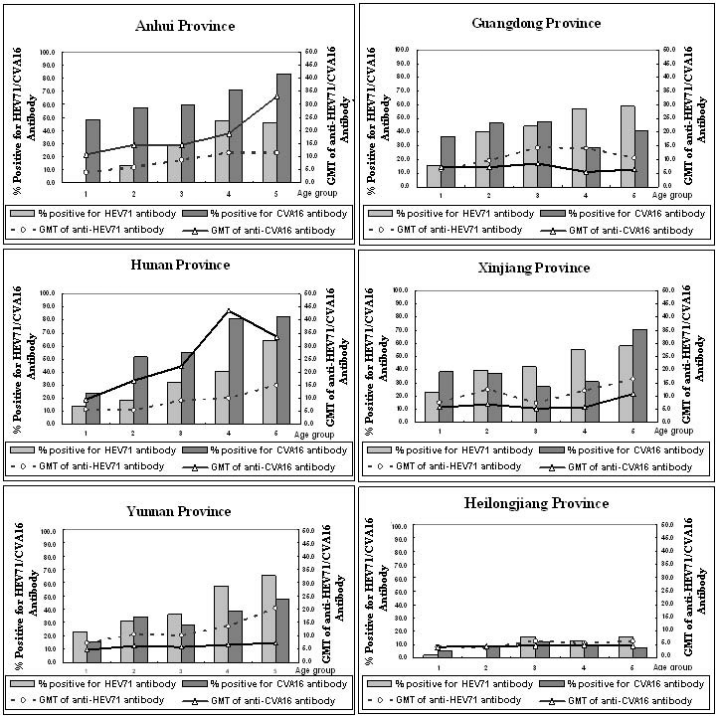
**Positive rates of neutralizing antibody and geometric mean titers (GMT) of human enterovirus 71 (HEV71) and coxsackievirus A16 (CVA16) in 6 provinces of China**.

For HEV71, the positive rates of neutralizing antibody and GMTs in Guangdong (43.7% and 1:10.8, respectively), Xinjiang (45.4% and 1:11.1, respectively), and Yunnan (43.4% and 1:12.0, respectively) provinces were relatively high, whereas the values were lowest in Heilongjiang province (8.1% and 1:4.9, respectively). For CVA16, the positive rates of neutralizing antibody and GMTs in Anhui (62.2% and 1:16.0, respectively) and Hunan (61.1% and 1:23.1, respectively) provinces were relatively high, whereas Heilongjiang province (8.0% and 1:4.6, respectively) had the lowest values (Figure 1).

There was an increasing tendency that the positive rate of HEV71 neutralization antibody increased with age among children aged 1-5 in Anhui, Hunan, Yunnan, Guangdong and Xinjiang provinces, and of which 3 provinces-Anhui, Hunan, and Yunnan- also appeared an similar increasing tendency about GMT of HEV71. For CVA16, both the positive rate of neutralization antibody and GMT appeared an increasing tendency with age among children aged 1-5 in Anhui and Hunan provinces (Figure 1).

There was a significant difference in the positive rates of neutralizing antibody of HEV71 and CVA16 among these 6 provinces (Chi-square test, HEV71: χ^2 ^= 63.1, P < 0.05; CVA16: χ^2 ^= 173.3, P < 0.05). And there was also a significant difference in the GMTs of HEV71 and CVA16 among these 6 provinces (Mann-Whitney U test, HEV71: P < 0.05; CVA16: P < 0.05).

### Low neutralizing antibody positive rate and titer of HEV71 and CVA16 in different geographical areas of China

**Figure 2:**
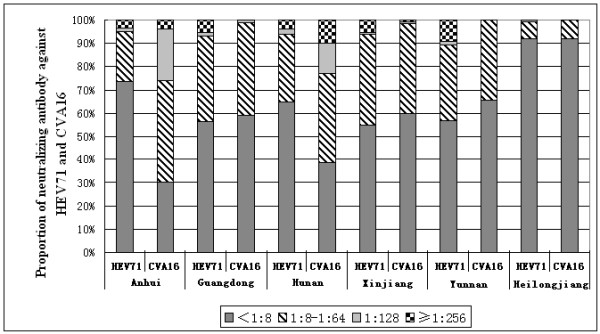
**Neutralizing antibody levels of HEV71 and CVA16 in 6 provinces of China**.

Among the 900 serum samples surveyed, the composition ratios for the neutralizing antibody titers of <1:8, 1:8-1:64, 1:128, and ≥1:256 were 68.0%, 26.4%, 1.3%, and 4.2%, respectively, for HEV71 and 56.6%, 37.6%, 2.9%, and 2.9%, respectively, for CVA16. All the studied provinces showed low neutralizing antibody positive rate and titer of HEV71 and CVA16, especially in Heilongjiang province, where the positive rate was 8.1% for both HEV71 and CVA16. All provinces except Heilongjiang showed ≥1:256 neutralizing antibody titers of HEV71 in 38 sera samples, and 3 provinces-Anhui, Hunan, and Xinjiang-showed ≥1:256 neutralizing antibody titers of CVA16 in 26 sera samples, indicating that HFMD infection occurred in 2005 (Figure 2).

## Discussion

The seroepidemiology of HFMD has not been well studied in China and in other countries; only a few studies on HEV71 have been conducted in Japan, Brazil, Singapore, and Taiwan [18-21]. HEV71 and CVA16 infections have been responsible for outbreaks and epidemic of HFMD, although 50-80% of the infections were asymptomatic.

A serologic survey would be useful to determine the transmission of virus in a natural setting. With the detection of neutralizing antibodies, a guide for future immunization programs against HFMD could be developed. This report can also provide scientific evidences for the development of prevention and control measures against HFMD in the future.

This is the first report that details the retrospective seroepidemiology of HEV71 and CVA16 in mainland China after the large-scale outbreaks occurred in 2008. The results showed a significant difference in the positive rates of neutralizing antibody and GMTs of HEV71 and CVA16 among 6 provinces in China, indicating a geographical difference in HEV71 and CVA16 infections. This research indicates that CVA16 infections occurred more frequently than HEV71 infections in east and central China, whereas HEV71 infections occurred more frequently than CVA16 infections in northwest, south, and southwest China. HEV71 and CVA16 infections were inactive in northeast China (Heilongjiang province), which may be due to the cold climate (average -14.7°C in winter season, and average 17°C in summer season), low population density (80.2 people per square kilometer in year 2010), a small number of children aged 1-5, and so on. Heilongjiang province has the lowest temperature in china, and usually human enteroviruses infection such as HFMD [13], aseptic meningitis [22], acute hemorrhagic conjunctivitis [23], and poliomyelitis, has peak incidence in summer season, that is to say, Heilongjiang province may have short time window to get more human enteroviruses infections.

Although there is a geographical difference in HEV71 and CVA16 infections in the past 5 years, low positive rate and titer of neutralizing antibody against HEV71 and CVA16 were found in all 6 provinces. More than 50% of children ≤5 years had no neutralizing antibody against HEV71 and CVA16. This led to accumulation of a large number of susceptible individuals, which may be partly responsible for the nationwide large outbreaks of HFMD caused by HEV71 and CAV16 in mainland China from 2008 [17]. During the big HFMD outbreak in Anhui province in 2008, another seroepidemiology survey was conducted, it showed that the positive rates of neutralizing antibody against HEV71 among the patients aged 1-5 were 22.5-66.7%, which is a substantial increase compared to the same indicator in 2005 (0-46.2%, Figure1) among the same age group.

The number of HFMD patients in all these 6 provinces reported by the notifiable infectious disease reporting system increased dramatically since HFMD was introduced as a category C notifiable infectious disease in China. And the numbers of HFMD patients of all these 6 provinces in 2009 were 1.28-2.61 times increasing in 2008, especially in Heilongjiang province where was low immunity level against HEV71 and CVA16 in 2005, a big HFMD outbreak attacked 36237 patients with 17 death in 2009, which is 2.61 times compared with the number of HFMD patients in 2008 (data from the notifiable infectious disease reporting system in China).

No HFMD surveillance data were available for the 6 provinces before 2008. This report confirmed that HEV71 and CVA16 had wildly circulated in mainland China before the large-scale outbreaks from 2008. This finding also suggests that public health measures to control the spread of HEV71 and CVA16 should be devised according to the different regional characteristics of mainland China.

## Methods

### Serum samples

The material used in this study is serum samples collected from the health children ≤5 years of age for the purpose of public health initiated by Chinese Ministry of Health, and the written informed consents from all participants (their parents) involved in this study were obtained for the use of their serum samples. This study has been approved by the second session of Ethics Review Committee in Chinese Centre for Disease Control and Prevention.

**Figure 3:**
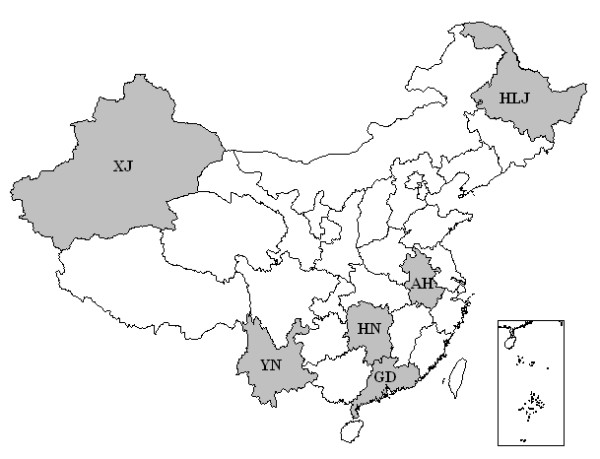
**Provinces from where serum samples were collected**. **Abbreviations of provinces of China: AH, Anhui; GD, Guangdong; HLJ, Heilongjiang; HN, Hunan; XJ, Xinjiang; YN, Yunnan**.

Nine hundred children ≤5 years of age were surveyed. Serum samples were collected randomly, with informed parental consent, in August 2005 by the Provincial Centers for Disease Control and Prevention in 6 provinces: 148 in Heilongjiang (northeast China), 130 in Xinjiang (northwest China), 250 in Anhui (east China), 131 in Hunan (central China), 119 in Guangdong (south China), and 122 in Yunnan (southwest China) (Figure 3). All children had no sign of disease at the time of sample collection.

The serum samples, which had been used in a previous study on measles, were divided and stored at -40°C.

### Neutralizing antibody detection

Neutralizing antibodies against HEV71 and CVA16 were detected with a neutralization test by microtechnique on human rhabdomyosarcoma (RD) cell line, as previously described with some modifications [18]. Serum samples were inactivated at 56°C for 30 min before use, and sample dilutions of 1:8 to 1:512 were assayed. Twenty-five microliters of virus, with a tissue culture infective dose (TCID_50_) of 100, was mixed with 25 μl of the appropriate serum dilution and incubated. For the serology results where GMT is reported, 1/2 positive critical value of antibody level was look upon as the antibody titer of the negative sera and calculated.

The HEV71 isolate (subgenotype C4a, GenBank accession number: EU703812,) used in this study was isolated from a patient with HFMD in Anhui province in 2008, while the CVA16 isolate (subgenotype B1b, GenBank accession number: GQ429229) was isolated from another patient with HFMD in Shandong province in 2007 [24].

An antibody titer of ≥8 was considered positive, and GMT was also calculated. Statistical analysis was carried out using SPSS version 13.0 software (SPSS Inc., Chicago, IL, USA), and Chi-square test was used to determine significance of neutralization antibody positive rates of HEV71 and CVA16, and Mann-Whitney test was used to determine significance of GMTs of HEV71 and CVA16 among these 6 provinces.

## List of abbreviations used

CVA16: coxsackievirus A16; GMT: geometric mean titer; HEV71: human enterovirus 71; HFMD: hand, foot, and mouth disease

## Competing interests

The authors declare that they have no competing interests.

## Authors' contributions

ZZ and WBX prepared manuscript. WBX designed the study and organized the coordination. ZZ, XBG and YZ performed data analysis. ZZ, SLZ, XBG, JTW, DYW, DMY, XJT, LYT, HZ, ZHY, XHJ and YXJ performed neutralization tests. All authors read and approved the final manuscript.

## References

[B1] Schmidt NJ Lennette EH Ho HH An apparently new enterovirus isolated from patients with disease of the central nervous system J Infect Dis 1974 129 304 309 4361245 10.1093/infdis/129.3.304

[B2] Li L He Y Yang H Zhu J Xu X Dong J Zhu Y Jin Q Genetic characteristics of human enterovirus 71 and coxsackievirus A16 circulating from 1999 to 2004 in Shenzhen, People's Republic of China J Clin Microbiol 2005 43 3835 3839 10.1128/JCM.43.8.3835-3839.2005 16081920 PMC1233905

[B3] Perera D Podin Y Akin W Tan CS Cardosa MJ Incorrect identification of recent Asian strains of Coxsackievirus A16 as human enterovirus 71: improved primers for the specific detection of human enterovirus 71 by RT PCR BMC Infect Dis 2004 4 11 10.1186/1471-2334-4-11 15122971 PMC415548

[B4] Chang LY Lin TY Huang YC Tsao KC Shih SR Kuo ML Ning HC Chung PW Kang CM Comparison of enterovirus 71 and coxsackie-virus A16 clinical illnesses during the Taiwan enterovirus epidemic, 1998 Pediatr Infect Dis J 1999 18 1092 1096 10.1097/00006454-199912000-00013 10608631

[B5] McMinn PC An overview of the evolution of enterovirus 71 and its clinical and public health significance FEMS Microbiol Rev 2002 26 91 107 10.1111/j.1574-6976.2002.tb00601.x 12007645

[B6] Chong CY Chan KP Shah VA Ng WY Lau G Teo TE Lai SH Ling AE Hand, foot and mouth disease in Singapore: a comparison of fatal and non-fatal cases Acta Paediatr 2003 92 1163 1169 10.1111/j.1651-2227.2003.tb02478.x 14632332

[B7] McMinn P Stratov I Nagarajan L Davis S Neurological manifestations of enterovirus 71 infection in children during an outbreak of hand, foot, and mouth disease in Western Australia Clin Infect Dis 2001 32 236 242 10.1086/318454 11170913

[B8] Shimizu H Utama A Yoshii K Yoshida H Yoneyama T Sinniah M Yusof MA Okuno Y Okabe N Shih SR Chen HY Wang GR Kao CL Chang KS Miyamura T Hagiwara A Enterovirus 71 from fatal and nonfatal cases of hand, foot and mouth disease epidemics in Malaysia, Japan and Taiwan in 1997-1998 Jpn J Infect Dis 1999 52 12 15 10808253

[B9] Jee YM Cheon DS Kim K Cho JH Chung YS Lee J Lee SH Park KS Lee JH Kim EC Chung HJ Kim DS Yoon JD Cho HW Genetic analysis of the VP1 region of human enterovirus 71 strains isolated in Korea during 2000 Arch Virol 2003 148 1735 1746 10.1007/s00705-003-0133-6 14505086

[B10] Chan LG Parashar UD Lye MS Ong FG Zaki SR Alexander JP Ho KK Han LL Pallansch MA Suleiman AB Jegathesan M Anderson LJ Deaths of children during an outbreak of hand, foot, and mouth disease in sarawak, malaysia: clinical and pathological characteristics of the disease. For the Outbreak Study Group Clin Infect Dis 2000 31 678 683 10.1086/314032 11017815

[B11] Fujimoto T Chikahira M Yoshida S Ebira H Hasegawa A Totsuka A Nishio O Outbreak of central nervous system disease associated with hand, foot, and mouth disease in Japan during the summer of 2000: detection and molecular epidemiology of enterovirus 71 Microbiol Immunol 2002 46 621 627 12437029 10.1111/j.1348-0421.2002.tb02743.x

[B12] Tu PV Thao NT Perera D Huu TK Tien NT Thuong TC How OM Cardosa MJ McMinn PC Epidemiologic and virologic investigation of hand, foot, and mouth disease, southern Vietnam, 2005 Emerg Infect Dis 2007 13 1733 1741 18217559 10.3201/eid1311.070632 PMC3375788

[B13] Zhang Y Tan XJ Wang HY Yan DM Zhu SL Wang DY Ji F Wang XJ Gao YJ Chen L An HQ Li DX Wang SW Xu AQ Wang ZJ Xu WB An outbreak of hand, foot, and mouth disease associated with subgenotype C4 of human enterovirus 71 in Shandong, China J Clin Virol 2009 44 262 267 10.1016/j.jcv.2009.02.002 19269888

[B14] Ho M Chen ER Hsu KH Twu SJ Chen KT Tsai SF Wang JR Shih SR An epidemic of enterovirus 71 infection in Taiwan. Taiwan Enterovirus Epidemic Working Group N Engl J Med 1999 341 929 935 10.1056/NEJM199909233411301 10498487

[B15] Liu CC Tseng HW Wang SM Wang JR Su IJ An outbreak of enterovirus 71 infection in Taiwan, 1998: epidemiologic and clinical manifestations J Clin Virol 2000 17 23 30 10.1016/S1386-6532(00)00068-8 10814935

[B16] Zheng ZM He PJ Caueffield D Neumann M Specter S Baker CC Bankowski MJ Enterovirus 71 isolated from China is serologically similar to the prototype E71 BrCr strain but differs in the 5'-noncoding region J Med Virol 1995 47 161 167 10.1002/jmv.1890470209 8830120

[B17] Zhang Y Zhu Z Yang WZ Ren J Tan XJ Wang Y Mao NY Xu ST Zhu SL Cui AL Zhang Y Yan DM Li Q Dong XP Zhang J Zhao YP Wan JF Feng ZJ Sun JL Wang SW Li DX Xu WB An emerging recombinant human enterovirus 71 responsible for the 2008 outbreak of hand foot and mouth disease in Fuyang city of China Virology journal 2010 7 94 10.1186/1743-422X-7-94 20459851 PMC2885340

[B18] Ooi EE Phoon MC Ishak B Chan SH Seroepidemiology of human enterovirus 71, Singapore Emerg Infect Dis 2002 8 995 997 12194783 10.3201/eid0809.10.3201/eid0809.010397 PMC2732542

[B19] Gomes Mde L de Castro CM Oliveira MJ da Silva EE Neutralizing antibodies to enterovirus 71 in Belem, Brazil Mem Inst Oswaldo Cruz 2002 97 47 49 11992146 10.1590/s0074-02762002000100006

[B20] Hagiwara A Tagaya I Komatsu T Seroepidemiology of enterovirus 71 among healthy children near Tokyo Microbiol Immunol 1979 23 121 124 223018 10.1111/j.1348-0421.1979.tb00448.x

[B21] Chang LY King CC Hsu KH Ning HC Tsao KC Li CC Huang YC Shih SR Chiou ST Chen PY Chang HJ Lin TY Risk factors of enterovirus 71 infection and associated hand, foot, and mouth disease/herpangina in children during an epidemic in Taiwan Pediatrics 2002 109 e88 10.1542/peds.109.6.e88 12042582

[B22] Mao NY Zhao LP Zhu Z Chen X Zhou SJ Zhang Y Cui AL Ji YX Xu ST Xu WB An aseptic meningitis outbreak caused by echovirus 6 in Anhui province, China Journal of medical virology 2010 82 441 445 10.1002/jmv.21707 20087933

[B23] Yan DM Zhu SL Zhang Y Zhang J Zhou YM Xu WB Outbreak of acute hemorrhagic conjunctivitis in Yunnan, People's Republic of China, 2007 Virology journal 2010 7 138 10.1186/1743-422X-7-138 20579343 PMC2901270

[B24] Zhang Y Wang DY Yan DM Zhu SL Liu JF Wang HY Zhao SC Yu DS Nan LJ An JJ Chen L An HQ Xu AQ Xu WB Molecular evidence of persistent epidemic and evolution of subgenotype B1 coxsackievirus A16-associated hand, foot, and mouth disease in China Journal of clinical microbiology 2010 48 619 622 10.1128/JCM.02338-09 20018819 PMC2815627

